# Emerging Challenges of Radiation-Associated Cardiovascular Dysfunction (RACVD) in Modern Radiation Oncology: Clinical Practice, Bench Investigation, and Multidisciplinary Care

**DOI:** 10.3389/fcvm.2020.00016

**Published:** 2020-02-21

**Authors:** Moon-Sing Lee, Dai-Wei Liu, Shih-Kai Hung, Chih-Chia Yu, Chen-Lin Chi, Wen-Yen Chiou, Liang-Cheng Chen, Ru-Inn Lin, Li-Wen Huang, Chia-Hui Chew, Feng-Chun Hsu, Michael W. Y. Chan, Hon-Yi Lin

**Affiliations:** ^1^Department of Radiation Oncology, Dalin Tzu Chi Hospital, Buddhist Tzu Chi Medical Foundation, Dalin, Taiwan; ^2^School of Medicine, Tzu Chi University, Hualien, Taiwan; ^3^Department of Radiation Oncology, Hualien Tzu Chi Hospital, Buddhist Tzu Chi Medical Foundation, Hualien, Taiwan; ^4^Cancer Centre, Dalin Tzu Chi Hospital, Buddhist Tzu Chi Medical Foundation, Dalin, Taiwan; ^5^Department of Biomedical Sciences, National Chung Cheng University, Chia-Yi, Taiwan; ^6^Department of Anatomic Pathology, Dalin Tzu Chi Hospital, Buddhist Tzu Chi Medical Foundation, Dalin, Taiwan

**Keywords:** radiation, cardiovascular dysfunction, miRNA, late sequelae, toxicity

## Abstract

Radiotherapy (RT) is a crucial treatment modality in managing cancer patients. However, irradiation dose sprinkling to tumor-adjacent normal tissues is unavoidable, generating treatment toxicities, such as radiation-associated cardiovascular dysfunction (RACVD), particularly for those patients with combined therapies or pre-existing adverse features/comorbidities. Radiation oncologists implement several efforts to decrease heart dose for reducing the risk of RACVD. Even applying the deep-inspiration breath-hold (DIBH) technique, the risk of RACVD is though reduced but still substantial. Besides, available clinical methods are limited for early detecting and managing RACVD. The present study reviewed emerging challenges of RACVD in modern radiation oncology, in terms of clinical practice, bench investigation, and multidisciplinary care. Several molecules are potential for serving as biomarkers and therapeutic targets. Of these, miRNAs, endogenous small non-coding RNAs that function in regulating gene expression, are of particular interest because low-dose irradiation, i.e., 200 mGy (one-tenth of conventional RT daily dose) induces early changes of pro-RACVD miRNA expression. Moreover, several miRNAs, e.g., miR-15b and miR21, involve in the development of RACVD, further demonstrating the potential bio-application in RACVD. Remarkably, many RACVDs are late RT sequelae, characterizing highly irreversible and progressively worse. Thus, multidisciplinary care from oncologists and cardiologists is crucial. Combined managements with commodities control (such as hypertension, hypercholesterolemia, and diabetes), smoking cessation, and close monitoring are recommended. Some agents show abilities for preventing and managing RACVD, such as statins and angiotensin-converting enzyme inhibitors (ACEIs); however, their real roles should be confirmed by further prospective trials.

## Introduction

Radiotherapy (RT) is an essential treatment modality in managing cancer patients (1, 2). Biologically, RT delivers ionizing radiation (IR) to eradicate cancer cells mainly through reacting with H_2_O to generate reactive oxygen species (ROS) to target multiple intra-cellular organelles, such as nucleus (mainly DNA), mitochondria, and cell membrane (3–5). Many IR-associated normal tissue damages are acute toxicities, characterizing potentially reversible and self-limited; however, some types of damages develop late sequelae, which are highly irreversible and progressively worse (4–6). For example, though the incidence is rare, irradiated cancer patients who had IR dose sprinkling to the cardiovascular system may encounter radiation-associated cardiovascular dysfunctions (RACVDs) (7–9), including blood pressure reduction (10), carotid stenosis (11), pericardial disease (12), myocardial infarction (13), pericardial/myocardial fibrosis (14, 15), valvular heart disease (16), arrhythmia (17), and subsequent heart failure (18–20). On clinical presentation, many RACVDs are late RT sequelae, developing a few years later after RT (21). Notably, as time elapsed, the risk of RACVD is larger in the third decade than that of the first two decades after IR exposure (22).

RACVD is a well-known treatment-related toxicity in the field of cardio-oncology (23–25). Other anti-cancer therapies, such as chemotherapy (26–29), targeted therapy (30–33), and immunotherapy (34–36), may also induce cardiovascular dysfunctions (37–39). As a result, when these therapies are prescribed concurrently or sequentially with RT, the risk of RACVD is increased substantially, especially in vulnerable pediatric (40, 41) or elderly cancer patients (42, 43). Besides, other RT-associated adverse events may occur with RACVD, such as ischemic stroke (44, 45) and lung fibrosis (46, 47), which may further impair patients' survival and life quality.

Several cardiovascular pathophysiological dysfunctions are associated with RT, such as late fibrosis/stenosis in the irradiated cardiovascular structures, mainly the endothelium (including endothelial cells and its stroma) and smooth muscle cells (2, 4, 5, 48). Epigenetic dysregulation, e.g., DNA methylation regulating gene expression without changes of sequence, demonstrates profound effects on the development of RACVD. For instance, differentially methylated enhancer of diacylglycerol kinase alpha (DGKA) reduces pro-fibrotic fibroblast activation, involving in radiation-associated tissue fibrosis and vascular stenosis (49). Similarly, microRNAs (miRNAs) also have been found to regulate the innate endothelium response to IR (50).

Clinically, moderate- to high-dose IR to the cardiovascular system increases the risk of RACVD (2, 4, 5). More notably, low-dose IR with a single 200 mGy (i.e., one-tenth of conventional RT daily dose of 200 cGy) has been observed to induce early damage of RACVD, demonstrating expression changes of miRNAs, e.g., miR-21 and miR-146b, and their regulated proteins in primary human coronary artery endothelial cells (51). This finding suggests that miRNAs as potential biomarkers for early detecting RACVD. Furthermore, some miRNAs have been reported as potential targets in managing RACVD, e.g., miR-15b (52), miR-21 (51–54), and miR-126-5p (55).

Hence, the present study aimed to review clinical challenges, potential biomarkers, and therapeutic targets of RACVD, with a focus on the role of miRNA. Emerging challenges of multidisciplinary care and example agents for prevention are also reviewed.

## Clinical Challenges and Emerging Issues for Detecting, Managing, and Preventing RACVD

### Clinical Challenges in Improving Detection, Management, and Prevention of RACVD

Several factors affect the risk of RACVD (Table 1). As a result, current treatment guidelines recommend several methods to reduce the risk of RACVD (1, 2, 56). For example, in patients at high risk, radiation oncologists always consider alternative treatment choice of deferred RT, adopt rigorous dose constraints on the cardiovascular system, or implement advanced irradiation techniques. However, even implementing advanced techniques, the occurrence of RACVD cannot be avoided totally. Several issues are still challenging in clinical practice.

**Table 1:** Factors affect the risk of RACVD.

**Factors**	**Description**	**References**
**PATIENT FACTOR**		
Pre-existing cardiovascular risk factors	Patients with pre-existing cardiovascular risk factors, such as prior cardiovascular disease, diabetes, COPD, smoking history, and high BMI (obesity), increase the risk of RACVD.	(21, 56–59)
BRCA1/2 mutation carriers	Patients with BRCA1/2 mutation demonstrate a higher risk of CVD than that of control patients.	(60)
Vulnerable populations	Pediatric and elderly cancer patients are vulnerable to RTCVD.	(40–43)
**CANCER FACTOR**		
Lung cancer	RT to lung cancers increases the risk of RTCVD.	(13, 61, 62)
Esophagus cancer	RT to esophagus cancers, especially the middle/lower third tumors, has a high risk of RTCVD.	(63)
Breast cancer	RT to breast cancers, especially the left side breast, burdens a substantial risk of RTCVD that may develop in decades.	(12, 21, 64, 65)
Head and neck cancers	RT to head and neck cancers increases the risk of RTCVD, mainly carotid stenosis and subsequent ischemic stroke.	(10, 45)
Lymphoma	RT to lymphomas that involved the thorax or head and neck regions demonstrates a high risk of RTCVD.	(8, 44, 66)
**RT HEART DOSE CONSTRAINS**		
^*^Lung SABR	**1. 50 Gy in 4 fractions:** V40 ≤ 1c.c.; V20 ≤ 5c.c.; Dmax ≤ 45 Gy. **2. 70 Gy in 10 fractions:** V45 ≤ 1c.c.; Dmax ≤ 60 Gy.	(67–69)
^*^Lung RT	V30 ≤ 45%; MHD < 26 Gy.	(67, 70)
^*^Breast RT	V5 < 10%; V25 < 5%; MHD <4 Gy.	(67)
^*^Esophagus RT	Dmax (0.03 cc) ≤ 52 Gy; V40 < 50%; MHD < 26 Gy.	(67)
^*^Lymphoma RT	MHD < 5 Gy ideal, no higher than 15 Gy.	(67)
**COMBINED THERAPY**		
^**^Chemotherapy	Some regimens demonstrate cardiotoxicities, e.g., anthracycline agents.	(26–29, 65)
^**^Targeted therapy	Some targeted therapy has cardiotoxicities, e.g., anti-Her2 and anti-VEGF agents.	(30–33)
^**^Immunotherapy	Some immunotherapeutic drugs have cardiotoxicities, e.g., anti-PD1/PDL1 agents.	(34–36)
**OTHER FACTORS**		
^***^Statins	Statins use may decrease the risk of RACVD in irradiated cancer patients.	(71)
^****^ACEI and angiotensin II receptor antagonist	These agents may decrease the risk of RACVD in irradiated cancer patients.	(72, 73)

* *The dose to OARs is different according to the irradiated sites and cancer disease extension. Radiation oncologists always judge the pros and cons of RT to achieve better tumor control and fewer toxicities, i.e., judging for maximum tolerated dose (MTD) or as low as reasonably achievable (ALARA) (67, 74)*.

** *Multimodality treatment is the cornerstone in managing cancer patients. However, combined treatments irreversibly enhance the risk of RTCVD*.

*** *Statin used in irradiated cancer patients with hypercholesterolemia may demonstrate double benefits of decreasing the blood level of cholesterol and the risk of RACVD*.

**** *ACEIs and angiotensin II receptor antagonists used in irradiated patients with hypertension may have double benefits of controlling blood pressure and decreasing the risk of RACVD*.

*“V5” represents the percent volume of organ at risk (i.e., the heart) that is irradiated with an IR dose of ≥ 5 Gy. V25, V30, V40, and V45 are similar representations*.

*ACEI, angiotensin-converting enzyme inhibitor; ALARA, as low as reasonably achievable; BMI, body mass index; COPD, chronic obstructive pulmonary disease; Dmax, maximal dose; Gy, gray; MHD, mean heart dose; MTD, maximum tolerated dose; OAR, organ at risk; RACVD, radiation-associated cardiovascular dysfunction; RT, radiotherapy; SABR, stereotactic ablative body radiotherapy; VEGF, vascular endothelial growth factor*.

#### Clinical Challenges of Decreasing the Risk of RACVD in Modern Radiation Oncology

Clinically, the overall incidence of RACVDs is rare but substantially encountered in irradiated patients with mediastinum lymphoma (8, 44, 66), head and neck (10, 45), esophagus (63), lung (13, 61, 62), and breast (12, 21, 56, 64) cancers. High-risk features of RACVD development are as follows: left-side breast irradiation (21, 65), combination with anthracycline-based chemotherapy (65), and vulnerable patient populations [e.g., pre-existing cardiac risk factors/heart disease (21, 57) and BRCA1/2 mutation carriers (60)]. For example, for a typical 50-year-old woman with pre-existing cardiac risk factors, an estimated 20-year risk of death from ischemic heart disease after breast RT is up to 1.6%, which is higher than that of those patients with no RT (i.e., 0.9%) (21, 56, 75). Moreover, in irradiated left breast cancer patients, each additional Gray (Gy) of the mean heart dose (MHD) increases the relative risk of major cardiac events by 7.4% (21).

Radiation oncologists implement several methods to decrease IR dose to the heart for minimizing the risk of RACVD (76), such as prone positioning (77), heart block with electronic compensation (57), heart-sparing three-dimensional printing technique (78), continuous positive airway pressure (CPAP) (79), real-time position management (RPM) inspiration gating (80, 81), proton-beam irradiation (82–85), and deep-inspiration breath-hold technique (DIBH) (86–90). However, even with the highly recommended visual-guided DIBH technique, residual variations of the heart position are still noticeable (91). As a result, the actual heart dose may be underestimated, burdening a higher risk of cardiac toxicities than that of estimation from the RT treatment planning.

For reducing the risk of cardiac toxicities, modern irradiation techniques aim to decrease irradiation dose to the heart. Diminishing the mean heart dose (MHD) is the main goal based on data estimated from conventional tangential technique (21, 92–94). Nevertheless, attenuating IR doses to coronary artery (95–97) and other cardiac substructures, such as left anterior descending artery (LAD) and left ventricle (LV), are more reasonable and suitable in modern precise RT departments (2, 66, 95, 98). However, long-term results investigated dose effects on these cardiac substructures are pending.

Another emerging challenge in clinical radiation oncology is the concept-shifting on treatment consideration. Previously, radiation oncologists always apply IR dose to organs at risk (OARs) according to the principle of “as low as reasonably achievable (ALARA) (99).” However, in some patient populations that required very aggressive managements, the treatment concept frequently shifts to maximum tolerated dose (MTD) for gaining the ultimate tumor control (67, 74). Undoubtedly, adopting MTD increases the heart dose and then burdens a higher risk of RTCVD than that of ALARA.

#### Challenges of Clinical Detection for RACVD

Early detection of RACVD is challenging. Some clinical predictors have been reported for stratifying patients at risk, such as dosimetric parameters of RT (61), cardiac risk index (100), and coronary calcium score (101). Moreover, biomarkers are clinically helpful for detecting RACVD (102), such as cardiac troponins (e.g., troponin I or T) and natriuretic peptides (e.g., B-type natriuretic peptide (BNP) or pro-BNP) (103). On imaging, echocardiography is the pivotal method to detect cardiac anatomic and functional changes of RACVD (104–106). Profound RACVD may show a reduction of LV ejection fraction, and subclinical disease may reveal early signs of decreased global longitudinal strain (107–109). Recently, other advanced imaging modalities are attractive for detecting RACVD (110), such as cardiac computed tomography (111–113) and cardiovascular magnetic resonance (CMR) (114–116).

In recent precision cardio-oncology, it is a promise direction that applies combined omic-data and metabolic-function nuclear images (117), such as single-photon emission computed tomography (SPECT) (118) and positron emission tomography (PET) (119–122). Of these, PET that demonstrated metabolic changes of the heart is the most expecting image marker for detecting RACVD. However, identifying suitable isotopes of PET for early detecting RACVD is still challenging.

#### Challenge of Clinical Managements for RACVD

Unfortunately, there is still no effective method to restore RT-associated late sequelae, including RACVD, because their disease courses are generally irreversible (2, 4, 6, 56). However, several pre-clinic studies have suggested potential targets for therapeutic interventions, such as HMGB1 (123) and miR-212 (124). Moreover, selective irradiation to the heart induces early overexpression of pro-hypertrophic miR-212, leading the miR-212 intervention as a reasonable approach for RACVD (124).

#### Clinical Prevention for RACVD and Future Challenges

Some clinical agents may be used to prevent the occurrence of RACVD. For instance, statins, HMG-CoA reductase inhibitors prescribed for managing hypercholesterolemia, significantly reduces the risk of stroke [hazard ratio (HR) = 0.68; 95% confidence interval (CI), 0.48–0.98; *P* = 0.0368] and demonstrates a trend to decrease the risk of RACVD (HR = 0.85; 95% CI, 0.69–1.04; *P* = 0.0811) in irradiated cancer patients (71).

The detailed mechanism of statin in protecting the cardiovascular system is unclear. Some potential mechanisms are proposed. Firstly, statin inhibits RhoA GTPase (125), which is essential to mediate the irradiation inhibition of endothelial cell migration (126–128). Secondly, statin decreases cardiac endothelial cell permeability via activating ERK5 (129). Thirdly, statin enhances the release of Nitric Oxide (NO), which is crucial for improving endothelial function via regulating miR-221/222 (130). Fourthly, statin diminishes IR-induced responses of cardiac Connexin-43 and miR-21 (53) that involves in the process of cardiac fibrosis (52).

Clinical strategies, such as close monitoring, smoking cessation (58, 131), prescribing angiotensin-converting enzyme inhibitors (ACEIs), and β-blockers, are useful to prevent anthracycline-associated cardiac toxicities (132, 133). In the literature, ACEIs also showed a potential for preventing RACVD. For example, Captopril, one of ACEIs prescribed for hypertension or heart failure, has been found to decrease pulmonary endothelial dysfunction in irradiated rats (72). Similarly, Candesartan, an Angiotensin II Receptor Antagonist, has been reported to reduce the risk of RACVD in left breast irradiated patients (73). Thus, a potential mechanism of ACEI for cardioprotection may be demarcated reasonably by inhibiting angiotensin II to decrease the expression of TGF-β, a well-known pro-fibrogenic factor of post-IR late fibrosis (134, 135). However, these methods required further data support to demarcate their real roles in preventing the development of RACVD.

#### Future Challenge: Mixed-Agent-Associated Cardiotoxicity in Combined Treatments

The major clinical problem is that many cancer patients were managed with multimodality treatments. As a result, the incidence of multi-treatment-associated CVDs, such as combined anthracycline-based chemotherapy and RT (136), is much higher than that of isolated RACVD. This phenomenon increases the difficulty of prevention and management, mostly requiring combined care from multidisciplinary team members, including radiation oncologists, medical oncologists, and cardiologists.

### Emerging Challenge of Bench Studies to Improve Early Detection, Management, and Prevention of RACVD, Focusing on the Role of miRNA in Acting as a Biomarker and Therapeutic Target

As mentioned above, in addition to currently clinical use biomarkers, such as cardiac troponins (e.g., troponin I) and natriuretic peptides (e.g., BNP) (103), several pre-clinical studies have been investigated to explore underlying mechanisms of RACVD, such as TGF-β and PPAR-α signaling pathways (137, 138), damage-associated molecular patterns (DAMPs) (139), and miRNA modulations (138). Of these, endogenous small non-coding miRNAs that function in regulating gene expression (140) grasp more interest in terms of biomarkers (141–143) and therapeutic targets (144–146) (Table 2).

**Table 2:** Examples of miRNAs involved in the process of RACVD that are potential for severing as biomarkers or therapeutic targets.

**miRNA**	**Description**	**References**
miR-1	1. miR-1 involved in cardiac hypertrophy. 2. IR decreased miR-1 in the rat myocardium. 3. HRW attenuated post-IR miR-1 decrease.	(52)
miR-15b	1. miR-15b showed anti-fibrotic, anti-hypertrophic, and anti-oxidative profiles. 2. IR decreased miR-15b value. 3. HRW restored miR-15b value.	(52)
miR-21	1. IR increases miR-21 expression in the irradiated rat hearts. 2. miR-21 involves in the process of cardiac fibrosis. 3. HRW diminishes post-IR myocardial miR-21 levels.	(52)
	4. Statins decrease IR-induced cardiac miR-21 response.	(53)
	5. A single low-dose 200 mGy induces expression changes of miR-21 and its modulated proteins in primary human coronary artery endothelial cells.	(51)
	6. On the contrast, miR-21 may play a cardioprotective role through Per2-dependent mechanisms.	(54)
miR-29b	miR-29b is one of pro-RACVD miRNAs.	(147)
miR-30	miR-30, miR-155, and miR-210 involve in the process of vascular calcification, which is one of the end events of RACVD that induces coronary artery stenosis and ischemic heart disease, via exosome delivery to vascular smooth muscle cells.	(148)
miR-34a	MIF inhibits miR-34a to protect from radiation-induced cardiomyocyte senescence via targeting SIRT1.	(149)
miR-126-5p	Applying miR-126-5p therapy represents a potential to improve endothelial recovery and prevent post-IR vascular re-stenosis.	(55)
miR-146a	At 24 h after 2-Gy IR, miR-146a is significantly overexpressed.	(150)
miR-146b	Low-dose IR with a single 200 mGy induces expression changes of miR-146b and its modulated proteins in primary human coronary artery endothelial cells.	(51)
miR-155	miR-30, miR-155, and miR-210 involve in the process of vascular calcification, which is one of the end events of RACVD that induces coronary artery stenosis and ischemic heart disease, via exosome delivery to vascular smooth muscle cells.	(148)
	At 2 h after 2-Gy IR, the level of miR-155 is decreased. At 24 h after 2-Gy IR, miR-155 is significantly overexpressed.	(150)
miR-210	miR-30, miR-155, and miR-210 involve in the process of vascular calcification, which is one of the end events of RACVD that induces coronary artery stenosis and ischemic heart disease, via exosome delivery to vascular smooth muscle cells.	(148)
miR-212	1. Selective irradiation to the heart induced overexpression of pro-hypertrophic miR-212. As a result, miR-212 is a potential therapeutic target.	(124)
miR-221	1. Statins conduct cardiovascular protection through enhancing the release of NO that is associated mainly with an improvement of endothelial function via regulating miR-221/222.	(130)
	2. At 2 h after 2-Gy IR, the expression of miR-221 is significantly increased.	(150)
miR-222	1. Statins conduct cardiovascular protection through enhancing the release of NO that is associated mainly with an improvement of endothelial function via regulating miR-221/222.	(130)
	2. At 2 h after 2-Gy IR, the expression of miR-222 is significantly increased. At 24 h after 2-Gy IR, miR-222 is significantly down-regulated.	(150)

*HRW, hydrogen-risk water (H_2_ water); IR, ionizing radiation; mGy, micro-Gray; MIF, macrophage migration inhibitory factor; NO, Nitric Oxide; RACVD, radiation-associated cardiovascular dysfunction*.

#### Emerging Challenges for Investigating Biological Mechanisms of RACVD

Detail mechanisms of RACVD are not well-recognized. Some potential mechanisms and pathways have been proposed. For example, IR may impair corin function and inhibit natriuretic peptides to accelerate senescence of cardiac and endothelial cells, contributing to the development of RACVD (151). Besides, several pathways have been identified with involvement into the process of RACVD, such as the 5-lipoxygenase (5-LO)/leukotriene pathway (152), the miRNA-34a/sirtuin-1 signaling pathway (149), the Reactive Oxygen Species (ROS)-mediated p16 pathway (153), and the TGF-β-associated signaling (154).

Moreover, some molecules may play roles in the process of RACVD, such as peroxisome proliferator-activated receptor gamma (PPAR-γ) (155), Growth differentiation factor 15 (GDF15) (153), and RhoA GTPase (125) that is essential to mediate the irradiation inhibition of endothelial cell migration. More recently, by using RNA-seq, differential gene-expression profiles have been identified in mice models, such as Nrf2, PDK1, and sirtuins (156). However, despite these lines of evidence, the whole picture of RACVD development is still not well-demarcated.

Another emerging challenge of investigating bio-mechanisms of RACVD comes from the difference of biological effects among different irradiation sources, e.g., proton vs. photon beams. Although proton and photon beams activate similar canonical radiation response pathways, distinct vascular genomic responses have been observed in the murine aorta (157). That is, models established according to photon radiation may not accurately predict the risk of RACVD associated with proton radiation.

#### Emerging Challenge of Bench Studies for Early Detecting and Managing RACVD, Focusing on the Example Role of miRNA

In the literature, many clinical studies assessed circulating miRNA levels in peripheral blood for diagnosing, predicting, and monitoring human diseases (158–163), including cardiac and vascular disease (CVD) (164–169). For example, the combination of miR-34a-5p and fibrinogen levels have been reported as a useful tool in differentiating pre-thrombotic status in patients with stable coronary artery disease (165). Moreover, the plasma expression level of miR-423-5p has been reported to serve as a promising biomarker for stratifying patients with coronary artery disease (168).

Similarly, several miRNAs have been found to involve in the process of RACVD (147, 170, 171). For example, via exosomes-based delivery to vascular smooth muscle cells, miR-30, miR-210, and miR-155 play roles in developing vascular calcification, which is one of the end events of RACVD that induces coronary artery stenosis and ischemic heart disease (148). Remarkably, IR-induced miRNAs expression behaves in a dose- and time-depended manner (150, 172). For instance, at 2 h after 2-Gy IR, the expression of miRNA-221 and miRNA-222 are significantly increased, but the level of miRNA-155 is decreased. On the other hand, at 24 h after 2-Gy IR, miRNA-146a and miRNA-155 are significantly overexpressed, but miRNA-222 is down-regulated (150). These patterns of miRNA expression changes require attention in further prospective studies that intend to demarcate the role of miRNAs in association with RACVD.

Although it requires further efforts to bridge miRNAs from bench to bedside, some miRNAs are attractive in early detecting and managing RACVD (124). For instance, applying miR-126-5p therapy potentially improves endothelial recovery and prevents post-irradiation vascular re-stenosis (55). Besides, inhibiting miR34a by macrophage migration inhibitory factor (MIF) has been reported to reduce radiation-induced cardiomyocyte senescence via targeting SIRT1, implicating a novel strategy for managing RACVD (149). Moreover, molecular hydrogen, i.e., hydrogen-rich water (HRW; H_2_ water), shows protective effects on IR-induced heart damage via regulating miRNA-1, -15b, and -21 (52).

In conjunction with miRNAs, circular RNAs (circRNAs) have been identified to involve in the regulatory network of the cardiovascular system. In biological function, circular RNAs may interact with RNA-binding proteins and act as miRNA sponges that inhibit the function of correspondingly matched miRNAs (173), demonstrating an ability for serving as novel biomarkers to early detect cardiovascular disease (174).

Applying circulating miRNA levels of peripheral blood is an immediately translatable mean for screening/monitoring RACVD. When researchers selected their miRNA targets by a literature review (such as targets that listed in the present study), miRNA database search, or miRNA-specific sequencing, they can subsequently conduct prospective clinical studies to validate their targets of interest under the pre-defined purpose of detecting, screening, or monitoring RACVD by using blood samples. However, testing details of circulating miRNAs (such as measuring methods, timing, and cut-off point values) are still required to be validated by prospectively clinical trials.

In the ClinicalTrials.gov (175), two actively recruiting trials integrate circulating miRNA as predicting biomarkers to detect RACVD in irradiated breast cancer patients, entitled: (1), Pre- or Postoperative Accelerated Radiotherapy (POP-ART; Identifier: NCT03783364) and (2), Breast Cancer and Cardiotoxicity Induced by Radiotherapy: the BACCARAT Study (Identifier: NCT02605512). Of these, the BACCARAT study investigates the role of several types of circulating biomarkers in detecting RACVD, including B-type natriuretic peptide, TGF-β1, and several miRNAs (e.g., miR-1, miR-34, miR-126, and miR-155). The results of the two trials are highly anticipated.

One potential limitation of applying miRNA in clinical practice is that the expression level of specific miRNAs would be varied in different tissues and testing time points. Therefore, the studies proceeding on the ClinicalTrials.gov may be very informative. Before the information of these clinical trials is available, in the authors' consensus opinion, integrating miRNAs as a component of circulating biomarkers for detecting RACVD may be critically considered in future clinical trials and practice that apply RT. Several measuring time points that similar to the protocol of the BACCARAT study are suggested as follows: before RT, the middle term of the RT course, and five time points after RT (i.e., 1 day, 6 months, 2, 5, and 10 years).

Why the time points of 2, 5, and 10 years should be considered testing and measuring? The main reason is that RACVD is a well-known RT toxicity; it characterizes not only acute cardiovascular damage but also late sequelae of cardiovascular dysfunction that may be encountered a few years or decades after RT (21, 56, 75). Thus, long-term series measuring (i.e., 2-, 5-, and 10-years after RT) of target miRNA levels is useful for early detecting and monitoring the occurrence and severity altering of RACVD.

#### Emerging Challenge: Novel Agents and Managements for Treating RACVD

As mentioned above, TGF-β-associated signaling gains a substantial interest in investigating the process of RACVD. For example, reducing irradiation-induced TGF-β1 production through blocking the NF-kB signaling pathway has been reported to provide a new insight in inhibiting irradiation-induced myocardial fibrosis (154). Besides, Protein Kinase C (PKC) has been reported to play a role in the process of RACVD (48). Remarkably, inhibiting PKC, such as applying RNA-interference techniques (176), could be a reasonable approach for managing IR-induced vascular dysfunction (48).

Some radioprotection agents, such as L-arginine, show protection effects on blood vessels of urinary bladder wall in patients treated with pelvic RT (177). Furthermore, IR-damaged vascular dysfunction has been observed to be restored by quercetin-filled phosphatidylcholine liposomes and mesenchymal stem cell injection (48). However, the real clinical roles of these agents and interventions on the cardiovascular system require further evidence to define.

### Emerging Challenge: Further Multidisciplinary Cooperation Among Radiation Oncologists, Cardiologists, and Molecular Biologists

Multidisciplinary care is required for preventing, detecting, and managing RACVD in irradiated cancer patients (178). In conjunction with the improvement of detection methods, increasing awareness and integrating works between oncologists and cardiologists are essential (179). Managing comorbidities adequately [e.g., hypertension, hypercholesterolemia, and diabetes control (180)], exercise therapy (181), and smoking cessation (58, 131) are all useful to decrease the risk of anti-cancer-treatment-related CVD (182), including RACVD. For multidisciplinary management, standard recommendation and structure/infra-structure requirements for patient care are ongoing established (183–188). For example, establishing consensus guidance to train RT staffs to delineate cardiac substructures decreases inter-observer variation and increases the accuracy of dose estimation, helping in implementing further randomized clinical trials and then daily clinical practice (189, 190).

Remarkably, several radiation-associated toxicities, including RACVDs, are diagnosed by a ruling-out—not ruling-in—way (2, 6). That is, diagnosing RACVD requires excluding other heart diseases, such as infectious disease or prior-existing subclinical cardiovascular dysfunctions. This work requires tight cooperation and interaction among multidisciplinary team members, such as radiation oncologists, medical oncologists, and cardiologists. Further consensus and recommendations are encouraged to establish in a multidisciplinary manner.

## Conclusion

Overall, the incidence of RACVD is rare in irradiated cancer patients. When it happened, however, RACVD may significantly impair patients' survival and life quality, particularly in vulnerable patient populations. Radiation oncologists implement many clinical efforts to reduce the risk; the incidence of RACVD is decreased but still substantial.

Further efforts from bench studies are emergently required to improve early detection, management, and prevention. For example, miRNAs play active roles in serving as biomarkers and therapeutic targets. Remarkably, integrating cooperation among multidisciplinary team members, such as oncologists and cardiologists, is encouraged and ongoing.

In the ClinicalTrials.gov (175), more than 20 clinical trials are actively or not yet recruiting for investigating challenging issues of RACVD, mainly focusing on early detection (e.g., circulating and imaging biomarkers) and aggressively avoidance/prevention (e.g., DIBH and proton therapy). Results from these ongoing trials are hopeful for resolving clinical obstacles of RACVD in the future.

## Author Contributions

All authors contributed to the brainstorming of the ideas generation. M-SL and D-WL also contributed to first draft writing. S-KH, C-CY, C-LC, W-YC, L-CC, R-IL, L-WH, C-HC, and F-CH also contributed to literature review and interpretation. H-YL and MC also co-corresponded to overall manuscript communication and final approval.

### Conflict of Interest

The authors declare that the research was conducted in the absence of any commercial or financial relationships that could be construed as a potential conflict of interest.
